# Data driven model of midportion achilles tendinopathy health created with factor analysis

**DOI:** 10.1186/s12891-022-05702-1

**Published:** 2022-08-03

**Authors:** Haraldur B. Sigurðsson, Christian Couppé, Karin Grävare Silbernagel

**Affiliations:** 1 Delaware Tendon Research Group, Department of Physical Therapy, University of Delaware, 540 S College Ave, Newark, Delaware 19713 USA; 2 School of Health Sciences, University of Iceland, Reykjavík, Iceland; 3 Department of Physical Therapy, Institute of Sports Medicine, Department of Orthopaedic Surgery M, Bispebjerg Hospital and Center for Healthy Aging, Faculty of Health and Medical Sciences, University of Copenhagen, Copenhagen, Denmark

**Keywords:** Health domains, Musculoskeletal health, Outcome measures, Metabolic syndrome, Ultrasound

## Abstract

**Background:**

Achilles tendinopathy is a complex injury and the clinical presentation spans multiple different domains: physical and psychological symptoms, lower extremity function and tendon structure. A conceptual model of Achilles tendon health comprising these domains has been proposed in the literature. The aim of the study was to fit a model of Achilles tendinopathy using factor analysis and compare that to the conceptual model. An inclusive approach using a wide range of variables spanning multiple potential domains were included.

**Methods:**

Participants (*N* = 99) with midportion Achilles tendinopathy were assessed with variables representing symptoms, physical function, tendon structure, metabolic syndrome, and psychologic symptoms. A Kaiser-Mayer-Olkin index was used to determine suitable variables for a subsequent exploratory factor analysis.

**Results:**

A model emerged with an acceptable fit to the data (standardized root mean square of residuals = 0.078). Five uncorrelated factors emerged from the model and were labelled as biopsychosocial, lower extremity function, body size, load tolerance, and tendon structure. The total explained variance was 0.51 with the five factors explaining 0.14, 0.12, 0.10, 0.08, and 0.07 respectively. The results differed from the conceptual model as the factors of psychological variables and metabolic variables did not emerge from the analysis.

**Conclusion:**

A data driven model of Achilles tendon health supports assessment of the clinical presentation over multiple domains. As the factors are uncorrelated, the results of assessment of, for example, tendon structure should not be expected to be associated with lower extremity function or biopsychosocial limitations. The results suggest that the Patient Reported Outcomes Measurement Information System, counter-movement jump height, body mass index, pain with hopping, and the tendon cross-sectional area can evaluate the five factors, respectively.

**Trial registration:**

Registered on clinicaltrials.gov (Medicine NL of. ClinicalTrials.gov [Internet], 2018), ID number NCT03523325.

**Supplementary Information:**

The online version contains supplementary material available at 10.1186/s12891-022-05702-1.

## Introduction

### Background

Achilles tendinopathy is a common overuse injury in sports [1] and represents 1.5 visits per 1000 in physicians general practice [2]. Exercise interventions have shown partially satisfactory results when assessed by patient reported outcomes as there are large variations in response to treatment [3, 4]. Despite this, few studies have examined variables that may explain the variability in treatment response. Reasons for the variability have been proposed to be due to sex or physical activity levels [5]. To further improve the outcome in all patients with Achilles tendinopathy there is a need to fully describe the patient presentation to identify individuals who might need special considerations or alternative treatments.

To meet this need, a conceptual model of Achilles tendinopathy has been proposed [6], composed of multiple discrete domains. The classical symptoms of Achilles tendinopathy [7] are included in the model, as well as structural alterations [8–11], and lower extremity functional deficits [12, 13]. There are good reasons to believe that the model domains are at least somewhat independent, meaning that changes in one domain (like decreased symptoms) do not necessitate that other domains (like tendon structure) have changed. It’s been shown that tendon structural alterations consistent with Achilles tendinopathy also occur in asymptomatic tendons [14]. Similarly, lower extremity functional deficits may persist after resolution of Achilles tendinopathy symptoms [15]. Taken together, these studies support a model structure of at least three domains.

A fourth and fifth potential domain may also exist. The fourth domain, included in the conceptual model of Silbernagel et al. [6], was the psychological effects of Achilles tendinopathy [16], whose relationship to physical symptoms or tendon structure is less investigated. The fifth potential domain is related to metabolic factors. Two recent reviews have indicated that hypercholesterolemia [17] and hyperglycemia [18] are risk factors for Achilles tendinopathy and there is evidence of a mechanistic link as hypercholesterolemia and hyperglycemia may affect tendon structure [19, 20]. Patients may present with varying levels of these metabolic variables, and this may be a domain of an Achilles tendinopathy model.

Finding which variables across the various domains are related to treatment success is like finding a needle in a haystack. A way to systematically select variables to further investigate is needed. Using factor analysis [21], the first step would be to test if the proposed model of Achilles tendon health is consistent with the data. The resulting factor structure would be the basis of an Achilles tendinopathy model, and a patient can be described along each of the factors of the model. The factor analysis can also identify variables best suited to describe each of the domains of the model. Those variables would then be considered as the primary candidates to test if they are associated with treatment success.

### Aims

The aim of this study was therefore to use an existing dataset with a range of variables representing the potential domains to fit a model of Achilles tendinopathy health, in order to profile the presentation of Achilles tendinopathy. The analysis will identify which factors are present in the data, what variables are important for each factor, and whether the factors are independent (uncorrelated) of each other. We hypothesize that a model will emerge that is consistent with the conceptual model previously proposed [6], with factors representing symptoms, psychological symptoms, lower extremity function, and tendon structure. We also hypothesize that an additional factor, metabolic variables, will emerge. The impact would be identifying variables that can define the clinical presentation and quantify the domains identified.

## Methods

This study was conducted using baseline data collected as part of an ongoing trial comparing the effects on an Achilles tendon exercise treatment between men and women. That study was a registered clinical trial (ClinicalTrials.gov identifier: NCT03523325 [22]). All subjects signed an informed consent, and all procedures were approved by University of Delaware’s institutional review board.

### Subjects

A total of 154 subjects were screened for inclusion in the ongoing trial, out of which 99 participants met the inclusion criteria. Inclusion criteria were age between 18 and 65 and a clinical diagnosis of midportion Achilles tendinopathy [23] made by study clinicians based on a history of pain with loading and tenderness to palpation [7]. Pain with loading refers to any pain or discomfort in the Achilles tendon during activities which involve any of the movements known to load the Achilles tendon [24], such as running, jumping, or other weight bearing plantarflexion activities. Subjects were excluded if they had a history of total Achilles tendon rupture, were currently receiving other treatments, were unable to perform the exercises due to other impairments, or if the source of pain was determined to be only insertional tendinopathy or bursitis. An ultrasound examination was available to clinicians to help make that determination. Subjects were not excluded if they had additional pathology such as bursitis if midportion Achilles tendinopathy was the main diagnosis. Subject characteristics are reported consistent with recommendations from a recent consensus statement [25] in Table 1.

**Table 1:** Subject characteristics (*n* = 99)

Demographics	Mean (SD) ^a^ Median (IQR) ^b^	
Age (years)	50 (18) ^b^	
Height (cm)	171 (8.6) ^a^	
Weight (kg)	85.0 (19.8) ^a^	
BMI (kg / m^2)	28.8 (6.3) ^a^	
BMI categories	28 normal weight (BMI < 25), 37 overweight (25 < BMI < 30), 34 obese (BMI > 30)	
Duration of symptoms (months)	10 (30) ^b^^,c^	
Physical activity scale ^d^	Previous Physical Activity Level (N)	Current Physical Activity level (n)
1 – Hardly any physical activity	1	4
2—Mostly sitting, sometimes a walk, easy gardening or similar tasks	5	6
3- Light physical exercise around 2–4 h a week, e.g. walks, fishing, dancing, ordinary gardening, including walks to and from shops	13	16
4—Moderate exercise 1–2 h a week, e.g. jogging, swimming, gymnastics, heavier gardening, home-repairing or easier physical activities more than 4 h a week	16	15
5 – Moderate exercise at least 3 h a week, e.g. tennis, swimming, jogging, etc	22	29
6—Hard or very hard regularly and several times a week, where the physical exertion is great, e.g. jogging, skiing	42	29
Previous tendon injuries	Number of individuals	
Pain/Stiffness	14	
Tendinitis	8	
Minor traumatic injury	4	
Previous medical Diagnoses	Number of Individuals	
Heart condition	7	
Hypertension	16	
Type-2 Diabetes	1	
Rheumatologic disease	2	
Thyroid disorder	9	
Other non-specified diagnoses	15	
Medications within the last 6 months	Number of Individuals	
Fluoroquinolones	6	
Oral corticosteroids	4	
Statins	11	

*SD* Standard deviation, *IQR* Interquartile range

^a^ Table gives mean and standard deviation

^b^ Table gives median and interquartile range

^c^ Note that duration of symptoms is exponentially distributed, 41 subjects report less than 3 months of symptoms, 28 between 3 and 6 months, and 14 more than 6 months

^d^ [60]

### Data collection procedure

#### Patient reported outcome measures

A variety of patient-reported outcome measures were used. The Victorian Institute of Sports Assessment-Achilles (VISA-A) [26] as the currently most commonly reported questionnaire for symptom severity. The Foot and Ankle Outcome Score (FAOS) sports and quality of life subscales [27] for limitations specifically related to the foot and ankle. The Patient Reported Outcomes Measurement Information System (PROMIS) subscales [28], which measures disability over a wide range of non-specific domains. For psychological effects specific to the pain experience, the Tampa Scale of Kinesiophobia (TSK) [29], the Pain Catastrophizing Scale (PCS) [30], and the Central Sensitization Index (CSI) were chosen.

#### Health markers

The following general health measurements were taken: Heart rate, and blood pressure were measured. As a marker for system accumulation of advanced glycation end products relevant for glucose homeostasis [31], skin autofluorescence [32] was measured using the AGE reader (Diagnoptics, Groningen). Readings from the AGE reader have been validated against a tissue biopsy [33] but the reliability has not been reported in the literature. In addition, height and weight were measured, and the BMI calculated from those values.

#### Achilles tendon ultrasound measures

The structural properties of the most symptomatic Achilles tendon were measured with an ultrasound examination (LOGIQ e with a 5–13 MHz transducer, GE Healthcare, Chicago, USA). The following measures were taken in B-mode at 10 MHz.: cross-sectional area and thickness at the thickest part of the tendon [8], thickness at a healthy tendon portion [1], length from calcaneus to the soleus muscle (panorama mode), and length from calcaneus to the gastrocnemius musculotendinous junction (between the medial and lateral gastrocnemius, panorama mode). Additionally, the thickness of the soleus muscle [13] and cross-sectional area of both the lateral and medial Gastrocnemius muscles [13] were measured with the ultrasound with both heads of the Gastrocnemius measured in a single panorama.

Mechanical properties of the Achilles tendon were measured with a second, dedicated ultrasound unit (SonixMDP Q + with L14-5/38, 5–15 MHz transducer, Ultrasonix, Vancouver, Canada) using continuous shear wave elastography (cSWE) at the thickest region of the tendon. The shear modulus [9] and viscosity [11] were calculated from the wave speed of tissue displacement along the tendon caused by an external actuator [11]. The reliability of the shear modulus and viscosity are 0.697 and 0.856, respectively [34]. All ultrasound measurements, including cSWE, were performed three times and the average used for analysis. Ultrasound examiners also evaluated the presence of (as Yes/No) bursitis, calcifications [35], and neovessels (using power doppler [10]) affecting the Achilles tendon.

#### Clinical examination

The following clinical evaluations of the Achilles tendon were additionally performed. Palpation of the Achilles tendon insertion to evaluate the presence of insertional symptoms (as any pain either present or absent). The Achilles tendon resting angles [36] with flexed and extended knees were then measured with a digital inclinometer. The pain pressure threshold was measured using a hand-held algometer (Somedic Senslab, Sösdala, Sweden) with a 1cm^2^ surface area that applied a point pressure (medial Gastrocnemius muscle) or a squeeze (Achilles tendon) at a fixed rate of increasing pressure until the first onset of a painful sensation. The pain pressure threshold was repeated three times, and the average was used.

#### Functional measures

The following battery of functional tests was used [12], on the most symptomatic leg. Three trials of a single-leg counter-movement jump with hands behind the back. Two trials of 20 consecutive hops with free hand placement. Subjects were given a demonstration of the hop performance and instructed to perform repeated hops similar to jumping rope, until given the stop command. Three trials of a single-leg drop counter-movement, with hands behind the back. For all jump and hop tests, flight time was measured with a MuscleLab (Ergotest Innovation, Stathelle) infrared light mat and used to calculate height and the average of the trials entered the analysis. If a subject attempted a hop or jump but was unable to complete the test, the performance was given a height of zero cm and included in the analysis. One trial of the heel-rise endurance test was performed with the same methods as a previous study [37] during which the maximum repetitions [37], maximum height, and total work [37] were measured using a linear encoder and the MuscleLab software (Ergotest Innovation, Stathelle). Pain in the Achilles tendon was recorded for all tests as the highest reported number on a 11-point numerical rating scale (0–10).

### Statistical analysis

Study data were collected and managed using REDCap electronic data capture tools hosted at the University of Delaware [38, 39]. REDCap (Research Electronic Data Capture) is a secure, web-based software platform designed to support data capture for research studies, providing 1) an intuitive interface for validated data capture; 2) audit trails for tracking data manipulation and export procedures; 3) automated export procedures for seamless data downloads to common statistical packages; and 4) procedures for data integration and interoperability with external sources.

A Kaiser-Mayer-Olkin index (KMO) [40] was used to assess whether the data set was suitable for a factor analysis and to select which variables were suitable for the exploratory factor analysis. Variables were stepwise excluded according to the lowest measure of sampling adequacy until a threshold of at least 0.6 was achieved for the remaining data set. Each excluded variable then entered a second KMO analysis step and included if the measure of sampling adequacy for the data set remained above 0.6. A “mediocre” [41] threshold for the measure of sampling adequacy was chosen due to the low number of observations available, and to include the maximum number of variables. All observations with a missing value for at least one variable were excluded from the analysis. This means that the final number of subjects is influenced by the results of the KMO analysis such that subjects with missing data are included in the analysis only if the variables for which data is missing is excluded from the analysis in the KMO analysis.

A mix of continuous, binary, and ordinal data entered the KMO analysis. A mixed correlation matrix was therefore calculated [42]. However, after the KMO analysis, all remaining variables were suitable for a regular correlation matrix which was calculated for the factor analysis.

To test if the data could be reduced to fewer variables with a factor analysis, we used the Bartlett´s test of sphericity [43]. The Bartlett´s test of sphericity tests if a correlation matrix is different from an identity matrix (all correlations equal to zero). A significance of 0.05 was used for the Bartlett´s test of sphericity.

A parallel analysis was used to determine the number of factors extracted using eigenvalues > 1 as the criteria. The exploratory factor analysis was performed using maximum likelihood method and the “oblimin” rotation. The model fit of the results were expressed as the standardized root mean square of the residuals (SRMS). The SRMS is a measure of model misfit (the difference between the suggested model, and the data) where lower numbers indicate a better fit. The SRMS performs well for models with few observations and many variables [44]. For similar conditions as the current study, a cut-off of 0.09 (where models with SRMS above 0.09 are rejected) for the SRMR minimized the combined risk of Type I and Type II errors in a simulation study [45]. An SRMR score below 0.09 will be described as acceptable for this study. All data processing was performed using the data.table package [46] for the R statistical language [47]. The factor analysis was performed with the psych package [42].

The ideal results from a factor analysis are interpretable results. There is currently no consensus for determining an appropriate loading cut-off to include a variable within a factor when interpreting the results. As we used variables that were not specifically designed to represent a latent factor, a loading cut-off of 0.3 was deemed appropriate to assign a variable to a factor. After the factor analysis, each factor was labeled according to our interpretation of the commonalities of the included variables.

## Results

### Variable selection

Variables excluded based on the KMO score were presence of calcifications, bursitis, insertional symptoms, pressure pain threshold (both Achilles and medial Gastrocnemius muscle), having bilateral symptoms, neovascularization, skin autofluorescence, previous tendon injuries, sex, use of statin medications, previous diagnosis of hypertension, resting angle with knee extended, and thickness of a healthy tendon portion. After excluding variables, the overall measure of sampling adequacy was 0.64. The Bartlett´s test of sphericity indicated that a factor analysis could be carried out with p < 0.00001. The parallel analysis supported extracting 5 factors with Eigenvalues of 6.1, 5.2, 2.9, 2.6 and 1.5.

### Factor analysis

**Fig. 1:**
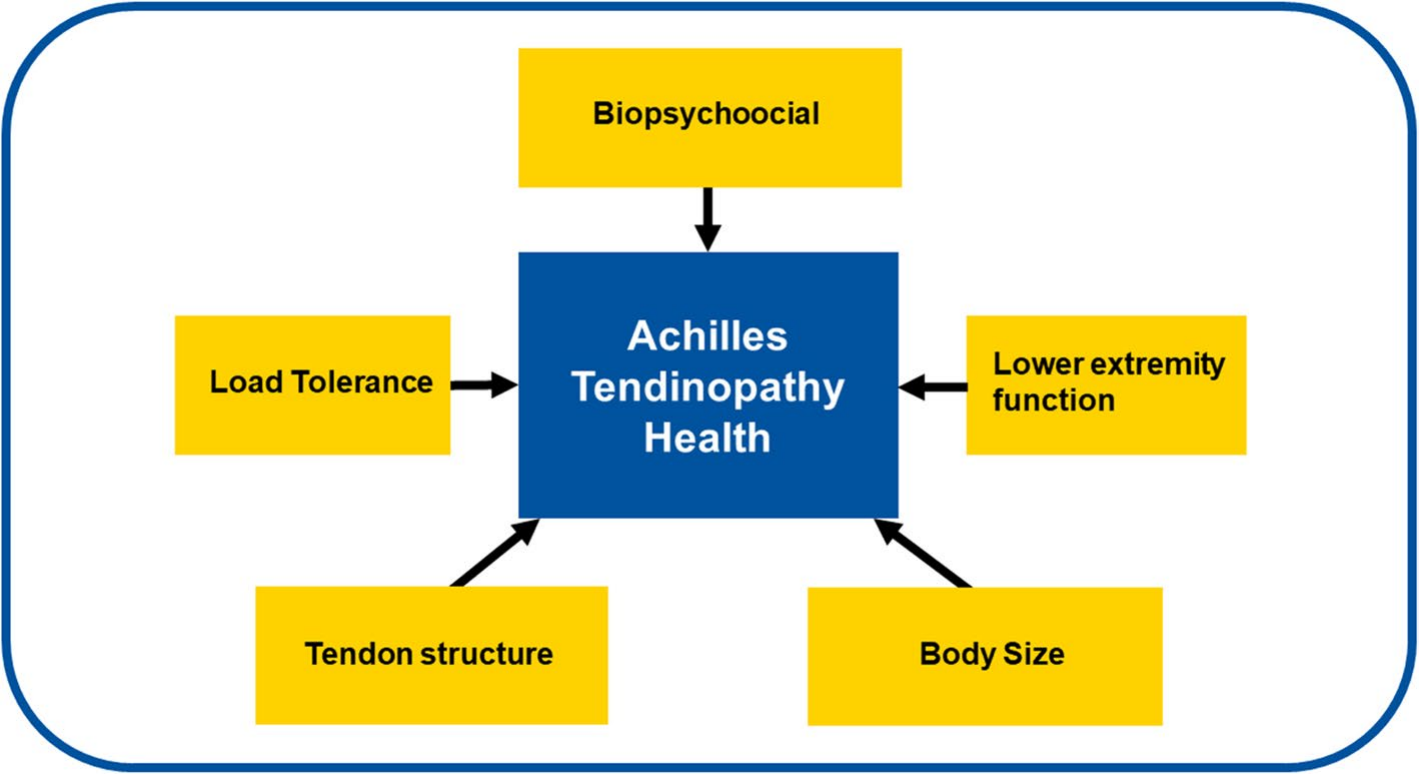
Visualization of the Achilles tendinopathy health model. The five domains that make up the model are represented by the outer boxes. The researcher-assigned factor labels are box headings

The five-factor model generated by the factor analysis with the researcher assigned labels is summarized in Fig. 1 and presented as an R readable file in Supplementary Information, Additional file 1 [2]. Factor loadings for each variable are reported in Tables 2, 3, 4,5 and 6 and summarized in Sect. 3.3. The variance explained by the factors were 0.14, 0.12, 0.10, 0.08, and 0.07 for factors 1:5 respectively. The cumulative variance explained was 0.51. The model fit was acceptable with an SRMS of 0.078. There were no correlations between any of the factors. Two variables that entered the analysis were not loaded into any factor (Table 7), thickness of the soleus muscle and tendon viscosity evaluated with cSWE.

**Table 2:** Loadings for variables in factor 1 – the symptom severity factor

	Factor labels				
	**Biopsychosocial**	Lower extremity function	Body size	Load tolerance	Tendon structure
Pain catastrophizing scale	0.83	0.07	0.04	0.03	-0.09
PROMIS Social roles & activities	-0.75	0.03	0.08	0.15	0.07
PROMIS anxiety	0.74	0.07	-0.09	-0.08	-0.03
PROMIS pain interference	0.71	-0.11	0.19	0.17	-0.02
PROMIS fatigue	0.66	-0.01	-0.23	-0.04	0.13
CSI	0.64	-0.11	-0.23	-0.02	0.02
PROMIS depression	0.64	-0.02	-0.10	-0.21	-0.11
FAOS quality of life	-0.60	-0.09	-0.11	-0.26	0.03
PROMIS sleep disturbances	0.60	-0.16	-0.03	-0.07	-0.01
FAOS Sports	-0.53	-0.11	-0.13	-0.20	0.04
TSK	0.51	0.11	0.28	-0.04	-0.06
VISA-A	-0.50	0.21	-0.01	-0.33	-0.06
PROMIS physical function	-0.47	0.11	-0.12	-0.24	-0.21
Achilles tendon resting angle with knee flexed	0.31	0.03	0.04	0.30	0.04

*Abbreviations*: *VISA-A* Victorian Institute of Sport Assessment for Achilles, *PROMIS* Patient reported outcomes measurement information system, *FAOS* Foot and Ankle Outcome Score, *CSI* Central sensitization index, *PCS* Pain catastrophizing index, *TSK* Tampa scale of kinesiophobia

**Table 3:** Loadings for variables in factor 2—the lower extremity function factor

	Factor labels				
	Biopsychosocial	**Lower extremity function**	Body size	Load tolerance	Tendon structure
Drop-CMJ height	0.01	0.89	0.018	-0.02	-0.07
CMJ height	-0.07	0.86	-0.06	0.04	-0.11
Hop height	-0.07	0.77	-0.14	-0.09	-0.01
HR work	-0.01	0.73	-0.01	0.14	0.02
HR reps	-0.01	0.51	-0.42	0.14	-0.02
Age	-0.11	-0.50	-0.10	-0.20	0.37
Height	0.04	0.49	0.43	-0.26	0.33
Gast length	-0.04	0.42	0.13	-0.17	0.31
Resting heart rate	-0.17	-0.38	0.12	0.05	-0.17
Physical activity level	-0.26	0.35	-0.24	0.12	-0.14

*CMJ* Counter-movement jump, *HR* Heel raise, *Gast* Length from Achilles tendon insertion to the Gastrocnemius muscle

**Table 4:** Loadings for variables in factor 3—the body size factor

	Factor Labels				
	Biopsychosocial	Lower extremity function	**Body size**	Load tolerance	Tendon structure
Weight	-0.04	-0.04	0.96	0.00	0.11
BMI	-0.06	-0.28	0.83	0.13	-0.08
Medial Gastrocnemius CSA	-0.03	0.10	0.76	0.03	-0.07
Lateral Gastrocnemius CSA	0.17	0.20	0.62	-0.19	0.04
Systolic blood pressure	0.04	-0.12	0.45	0.02	0.03
Heel raise pain	-0.09	-0.11	0.35	0.27	-0.20
Diastolic blood pressure	0.03	-0.23	0.33	0.02	0.02

*Abbreviations BM* Body Mass Index. *CSA* Cross sectional area, *Pain* Numerical rating scale (0–10)

**Table 5:** Loadings for variables in factor 4—the load tolerance factor

	Factor Labels				
	Biopsychosocial	Lower extremity function	**Body size**	Load tolerance	Tendon structure
Weight	-0.04	-0.04	0.96	0.00	0.11
BMI	-0.06	-0.28	0.83	0.13	-0.08
Medial Gastrocnemius CSA	-0.03	0.10	0.76	0.03	-0.07
Lateral Gastrocnemius CSA	0.17	0.20	0.62	-0.19	0.04
Systolic blood pressure	0.04	-0.12	0.45	0.02	0.03
Heel raise pain	-0.09	-0.11	0.35	0.27	-0.20
Diastolic blood pressure	0.03	-0.23	0.33	0.02	0.02

*Abbreviations: BMI* Body Mass Index. *CSA* Cross sectional area, *Pain* Numerical rating scale (0–10)

**Table 6:** Loadings for variables in factor 5—the tendon structure factor

	Factor labels				
	Biopsychosocial	Lower extremity function	Body size	**Load tolerance**	Tendon structure
Hop Pain	-0.03	0.07	-0.04	0.97	0.02
CMJ Pain	0.01	-0.04	-0.02	0.85	0.08
Drop-CMJ Pain	0.05	0.04	0.13	0.77	-0.05

*Abbreviations*: *Hop* 20 consecutive hop test, *CMJ* Counter-movement jump, *Drop-CMJ* Drop counter-movement jump, *Pain* Pain on a numerical rating scale (0–10)

**Table 7:** Loadings for variables that did not load highly on any factor

	Factor labels				
	Biopsychosocial	Lower extremity function	Body size	Load tolerance	Tendon structure
Thickness of the soleus muscle	0.10	-0.11	0.21	0.15	-0.20
Viscosity	0.30^a^	-0.03	-0.05	-0.21	-0.23

^a^ Rounded up from 0.297

### Factor labelling

The first factor was labelled as the biopsychosocial factor (Table 2, explained variance 0.14). The factor included the pain catastrophizing scale, the central sensitization index, all PROMIS subscales, the FAOS quality of life and sports subscales, TSK, the VISA-A, and the Achilles tendon resting angle with knee flexed.

The second factor was labelled as the lower extremity function factor (Table 3, explained variance 0.12). Included variables were height on all jumps and hopping measures, the heel-rise reps and heel rise work, subject age, height, length of Achilles tendon (gastrocnemius to calcaneus), resting heart rate and current physical activity level.

The third factor was labelled as body size (Table 4, explained variance 0.10). Included variables were weight, BMI, cross sectional area of both Gastrocnemius muscle heads, systolic blood pressure, pain during the heel-rises, and diastolic blood pressure. Secondary variables (variables that load above threshold, but load higher in another factor) were heel rise repetitions and height,

The fourth factor (Table 5, explained variance 0.08) was labelled as the load tolerance factor, and included pain reported during jump and hopping tests. The VISA-A was a secondary variable in load tolerance.

The fifth factor was labelled as the tendon structure factor (Table 6, explained variance 0.07). Included variables were thickness of the thickest portion of the tendon, tendon cross sectional area, length of Achilles tendon from soleus to calcaneus, and the tendon shear modulus. Secondary variables were age, length of the Achilles tendon from gastrocnemius to calcaneus and height.

## Discussion

### Summary

This is the first study to create a statistical model of a suggested Achilles tendinopathy health model [6]. The primary hypothesis of a five-factor model structure was only partially supported. Out of the hypothesized factors, symptom severity, tendon structure and lower extremity function were distinguishable. The hypothesized psychological symptom factor did not emerge, and all psychological variables were included in symptom severity. The hypothesized metabolic variables factor did not emerge, however a factor representing body size which included diastolic and systolic blood pressure did. An unexpected factor, load tolerance, emerged. The secondary hypothesis was supported, as the factors were uncorrelated and therefore represent independent domains of tendon health.

### Deviations from expected model

The resulting model deviated in three important ways from the conceptual model. Firstly, there was no factor representing symptom severity, but this was replaced by the biopsychosocial factor. This factor was composed of the patient reported outcome measures and included all of the questionnaires administered in the study. The VISA-A loaded on the lower-end of this factor and was also included in the load tolerance factor. The VISA-A questionnaire has previously been reported to evaluate more than one factor which could explain why it was included in two factors. A sensitivity analysis was conducted with each VISA-A factor entering the analysis as separate variables, but the interpretability of the results was unchanged (see Supplementary Information, Additional file 3). A recent study has questioned the content and construct validity of the VISA-A that also exhibits differential item functioning (DIF) depending on the duration period of symptoms [48]. Our results show that the VISA-A may not be sufficient to evaluate the biopsychosocial domain. Unexpectedly, the pain catastrophizing scale was the highest loading variable on the biopsychosocial factor. Pain catastrophizing has not previously been described as important in this patient population, however a recent study has concluded that a sub-group of patients with Achilles tendinopathy can be described as ‘psychosocial-dominant’ – identified as having the worst symptoms and highest degree of psychological effects but minimal tendon structure changes [49]. Pain catastrophizing may be very important in this subgroup which would explain the high loading for pain catastrophizing on the factor.

Secondly, no factor emerged for metabolic variables, potentially since no direct measure was included for hyperglycemia or hypercholesterolemia. Body size emerged instead, with BMI, weight, blood pressure and calf muscle cross-sectional area. Considering that BMI is an important metabolic variable, and that blood pressure loaded on this factor as well it can certainly be argued that the factor of metabolic variables still emerged. However, since weight loaded higher than BMI and gastrocnemius muscle CSA higher than blood pressure, we considered the label body size to be a better fit. There are good reasons to think that hypercholesterolemia [17, 50] and hyperglycemia [18] are clinically relevant for Achilles tendinopathy. While the healthy Achilles tendon undergoes virtually no remodeling after skeletal maturity [51], symptomatic Achilles tendinopathy has likely undergone much more collagen turnover [52]. In the remodeling period the tendinopathic Achilles tendon may accumulate advanced glycation end-products which impact tissue remodeling [53]. Skin autofluorescence, as a proxy for tendon autofluorescence, could in theory have loaded on either tendon structure or with metabolic variables. Similarly, hypercholesterolemia can result in accumulation of xanthomas in the Achilles tendon [19] which could affect measures of tendon structure. However, neither skin autofluorescence nor statin medication use entered the factor analysis. Blood pressure was the only variable that entered the factor analysis out of those selected as potential metabolic variables. The relationship between BMI and blood pressure is well documented [54], and blood pressure therefore fits well in a body size factor. However, blood pressure can also be an indicator for other metabolic variables [55]. It may be that more direct measures are required. Serum cholesterol and glycated hemoglobin may be sufficient for the metabolic variables factor to emerge, but direct measures of tendon metabolic variables (such as tendon glycation) are difficult to measure.

Thirdly, the factor of load tolerance was not expected to emerge from the analysis, but pain with loading is a hallmark of Achilles tendinopathy and was expected to be part of a symptoms factor. A recent study suggested that pain with loading is independent from VISA-A scores [56], and our results expand on that result by establishing pain with loading as an independent factor of the clinical presentation. Pain on the 20 consecutive hops test was the highest loading variable on this factor. The 20 consecutive hops test challenges the ability of the Achilles tendon to withstand repeated high loads and has been reported as the loading test that best differentiates subjects with Achilles tendinopathy from controls [12].

### Clinical utility

Our results show that clinicians can evaluate some facet of the five factors in the model to form a holistic assessment of the presentation of Achilles tendinopathy. A recommendation is made below based on the factor loadings from our results, but the precision of estimates for factor loading is likely poor. The clinician should therefore use their best judgement for what specific variables to use to represent the factors.

To evaluate the biopsychosocial aspect, our results support the use of the PROMIS subscales which have been validated for various chronic conditions [57]. For specific subscales, the highest loadings were for the ability to participate in social roles and activities, pain, and anxiety subscales. For lower extremity function, we recommend the single-leg counter-movement jump as a general measure. The single-leg drop counter-movement jump had slightly higher loading but has a lower completion rate limiting its usefulness for patients with lower functional capacity. The heel-rise test may be more desirable for patients who are unable to jump. As the difference in factor loadings between the three tests are small, the appropriate test can be chosen for each individual subject.

For load tolerance, pain from the hopping test was the best variable. To evaluate the tendon structure, the best variable is tendon cross-sectional area measured with ultrasound. To evaluate body size, weight had the highest loading, but BMI is perhaps more useful and therefore the best variable. Higher BMI may require greater adjustment of daily activities, as heel rise pain was also loaded on body size and heel rises are close in intensity to that of daily activities.

### Limitations

This study has several limitations to consider. An important limitation is that of sample size. A popular rule of thumb for the number of observations per variable in the factor analysis is 10:1, which would require 420 subjects in the analysis. However, such rules of thumb are without empirical support. A simulation study has been performed to analyze the required sample sizes under various ratios of observations, factors, and variables [58]. It was found that under the conditions closest to our study (5 factors, 30 variables, 90 subjects and wide communalities [58]), the per element accuracy was 96%, indicating that the majority of the variables under these simulation conditions were factored correctly. The ratio of variables to factors was higher in our study, which increases accuracy, but the ratio of observations to variables was lower which decreases accuracy.

The model fit was adequate as determined by the SRMS of 0.078. While below the 0.09 cut-off suggested to minimize the error rate [45], it is challenging to determine if the results are precise enough to rule out a deviation which would significantly change the interpretation of the results. Variables which load on more than one factor increase the model misfit as evaluated by the SRMS. In a sensitivity analysis, we repeated the factor analysis except variables that load on more than one value were excluded (age, height, length of Achilles tendon to gastrocnemius, and the heel rise repetitions). The SRMS value for this model was 0.058—indicating closer fit to the data (see Supplementary Information, Additional file 2). However, the interpretation of the results, and the labelling of the factors, remained unchanged.

The final number of subjects included in the factor analysis was 74. It’s been shown that even with small sample sizes, the factor structure of data can still be extracted [59]. The imprecision due to a smaller sample size in this study is likely to be in the estimations of individual variable loadings on the factors rather than in the factor structure. As the main purpose of this study was to identify the factor structure rather than the precise variable loadings, the sample size used is likely adequate.

Factor analysis results are sensitive to the variables input into them, and the choice to include or omit a variable has implications for the results. This was addressed using the KMO score for variable selection with the intent of making the results less subjective and more repeatable.

There may be additional factors we could not identify such as the metabolic factor. As an inherently exploratory approach, this study may be considered the first step towards creating a robust model. As with all exploratory analyses in science, results should be taken with a grain of salt until they are replicated. The ideal replication is a primary study with sufficient statistical power, but this approach is unlikely to be performed due to the expensive and time consuming data collection required. As an alternative to a study with hundreds of participants, a more practical approach would be to replicate the analysis with existing data from other clinical trials.

The study population in the current study is mixed. This is both a strength and a weakness. Many studies in Achilles tendinopathy focus on athletes or other individuals with high physical activity levels. However, a significant portion of people with Achilles tendinopathy do not fit the classical athletic overuse model. Our results should rather be generalized to the older person with more comorbidities rather than the younger athlete with Achilles tendinopathy. Many of our subjects have only experienced symptoms for a relatively short time and can be considered as more of an acute presentation. However, it’s likely that the pathological processes of tendinopathy precede the onset of symptoms by as much as several years [52]. The duration of symptoms is therefore perhaps not be very useful but this is none the less a potential limitation.

## Conclusions

This is the first data-driven study to suggest that five separate domains exist as a model of Achilles tendinopathy health. The factors may form a basis to describe the clinical presentation of a subject. Based on our results, we can suggest variables representative of each factor. For the biopsychosocial, we suggest the PROMIS subscales. For lower extremity function the counter-movement jump is suggested. BMI is suggested for body size. Pain during hopping is suggested for load tolerance. To represent tendon structure, the cross-sectional area of the thickest portion of the tendon is suggested.

## Supplementary Information

**Additional file 1.**

**Additional file 2.**

**Additional file 3.**

## Data Availability

The dataset analyzed during the current study is available from the corresponding author on reasonable request.
